# Elucidating the role of gut microbiota metabolites in diabetes by employing network pharmacology

**DOI:** 10.1186/s10020-024-01033-0

**Published:** 2024-12-20

**Authors:** Weiguo Yao, Jinlin Huo, Jing Ji, Kun liu, Pengyu Tao

**Affiliations:** 1 Department of Nephrology, Jinshan District Central Hospital, Shanghai University of Medicine & Health Sciences, Shanghai, China; 2 Institute of Precision Medicine, The First Affiliated Hospital of Shantou University Medical College, Shantou, China; 3 Department of Emergency, Shanghai Municipal Hospital of Traditional Chinese Medicine, Shanghai University of Traditional Chinese Medicine, Shanghai, China; 4 Department of Nephrology, Seventh People’s Hospital, Shanghai University of Traditional Chinese Medicine, Shanghai, China

**Keywords:** Diabetes, Gut microbiota, Metabolites, Network pharmacology, Kidney disease

## Abstract

**Background:**

Extensive research has underscored the criticality of preserving diversity and equilibrium within the gut microbiota for optimal human health. However, the precise mechanisms by which the metabolites and targets of the gut microbiota exert their effects remain largely unexplored. This study utilizes a network pharmacology methodology to elucidate the intricate interplay between the microbiota, metabolites, and targets in the context of DM, thereby facilitating a more comprehensive comprehension of this multifaceted disease.

**Methods:**

In this study, we initially extracted metabolite information of gut microbiota metabolites from the gutMGene database. Subsequently, we employed the SEA and STP databases to discern targets that are intricately associated with these metabolites. Furthermore, we leveraged prominent databases such as Genecard, DisGeNET, and OMIM to identify targets related to diabetes. A protein-protein interaction (PPI) network was established to screen core targets. Additionally, we conducted comprehensive GO and KEGG enrichment analyses utilizing the DAVID database. Moreover, a network illustrating the relationship among microbiota-substrate-metabolite-target was established.

**Results:**

We identified a total of 48 overlapping targets between gut microbiota metabolites and diabetes. Subsequently, we selected IL6, AKT1 and PPARG as core targets for the treatment of diabetes. Through the construction of the MSMT comprehensive network, we discovered that the three core targets exert therapeutic effects on diabetes through interactions with 8 metabolites, 3 substrates, and 5 gut microbiota. Additionally, GO analysis revealed that gut microbiota metabolites primarily regulate oxidative stress, inflammation and cell proliferation. KEGG analysis results indicated that IL-17, PI3K/AKT, HIF-1, and VEGF are the main signaling pathways involved in DM.

**Conclusion:**

Gut microbiota metabolites primarily exert their therapeutic effects on diabetes through the IL6, AKT1, and PPARG targets. The mechanisms of gut microbiota metabolites regulating DM might involve signaling pathways such as IL-17 pathways, HIF-1 pathways and VEGF pathways.

## Introduction

Diabetes Mellitus (DM) has become a major public health challenge faced by the world in the 21st century (Wang et al. 2023). The number of diabetes patients worldwide is expected to reach to 693 million by 2045, and this growth trend will produce profound impacts on human health, economy, and social development (Poulsen et al. 2023). Diabetes is a metabolic disorder caused by insufficient insulin secretion or impaired insulin action in the body. Prolonged high blood glucose levels can lead to organ damage, especially in the eyes, kidneys, nervous system, and cardiovascular system (Korbut et al. 2023). Diabetic Nephropathy (DN), as one of the main microvascular complications of diabetes, is an important cause of chronic kidney failure and end-stage renal disease (Dragoș et al. 2020). However, the pathogenesis of diabetes and diabetic nephropathy is still not fully elucidated. Consequently, there is an urgent need to investigate the pathogenesis of these conditions and identify more efficacious strategies for prevention and treatment.

Numerous studies have provided compelling evidence regarding the pivotal role of gut microbiota in human health (Saranya and Viswanathan 2023; Tain and Hsu 2023). Notably, some investigations have highlighted the beneficial influence of the dominant microbial communities, namely the phyla Firmicutes and Bacteroidetes, on overall health (Glorieux et al. 2023). These microbial populations exhibit a remarkable degree of stability and diversity in their distribution, thereby playing a critical role in maintaining the delicate equilibrium of the gut microbiota(Chen et al. 2023a, b). Importantly, under conditions of homeostasis, these microbial communities actively contribute to the preservation of a stable gut environment through intricate mechanisms, including the regulation of intestinal permeability, the modulation of inflammation and the secretion of short-chain fatty acids (SCFAs) (Zou et al. 2022; Abdolmaleky et al. 2023). The metabolites derived from gut microbiota play an important role in the development of diabetes. Some harmful metabolites, such as Trimethylamine oxide and lipopolysaccharides, have a negative impact on diabetes (Wu et al. 2023a, b). But SCFAs are beneficial metabolites derived from gut microbiota that regulate glucose and lipid metabolism through G-protein-coupled receptors, thereby improving insulin sensitivity, mitigating inflammatory responses and balancing energy homeostasis (Wu et al. 2023a, b; Tao et al. 2022). They exhibit a great potential therapeutic efficacy in the prevention and treatment of diabetes. Consequently, these multifaceted actions exert a profound positive impact on the host, further emphasizing the significance of these microbial communities in human health and disease.

Due to gut microbiota possessing remarkable efficacy and minimal adverse effects, it can act as a promising therapeutic approach for the treatment of diabetes (Hayeeawaema et al. 2023). The metabolites generated by the gut microbial community have been substantiated to exhibit positive therapeutic effects in the management of diabetes (Zhou et al. 2022). However, a comprehensive understanding of the active metabolites derived from the gut microbiota and their underlying pharmacological mechanisms in the context of diabetes requires further investigation. Recently, network pharmacology analysis, an interdisciplinary field that studies how drugs interact within complex biological networks rather than focusing solely on individual molecular targets, analysis has been recommended for exploring complex disease mechanisms. The active components and their associated targets within the disease network can be rapidly screened and identified through network pharmacology. In this study, we are going to employ the network pharmacology to uncover the transformation process and molecular mechanism of gut microbiota metabolites at the onset of DM.

## Methods and materials

### Identification of metabolites and targets of gut microbiota

**Fig. 1:**
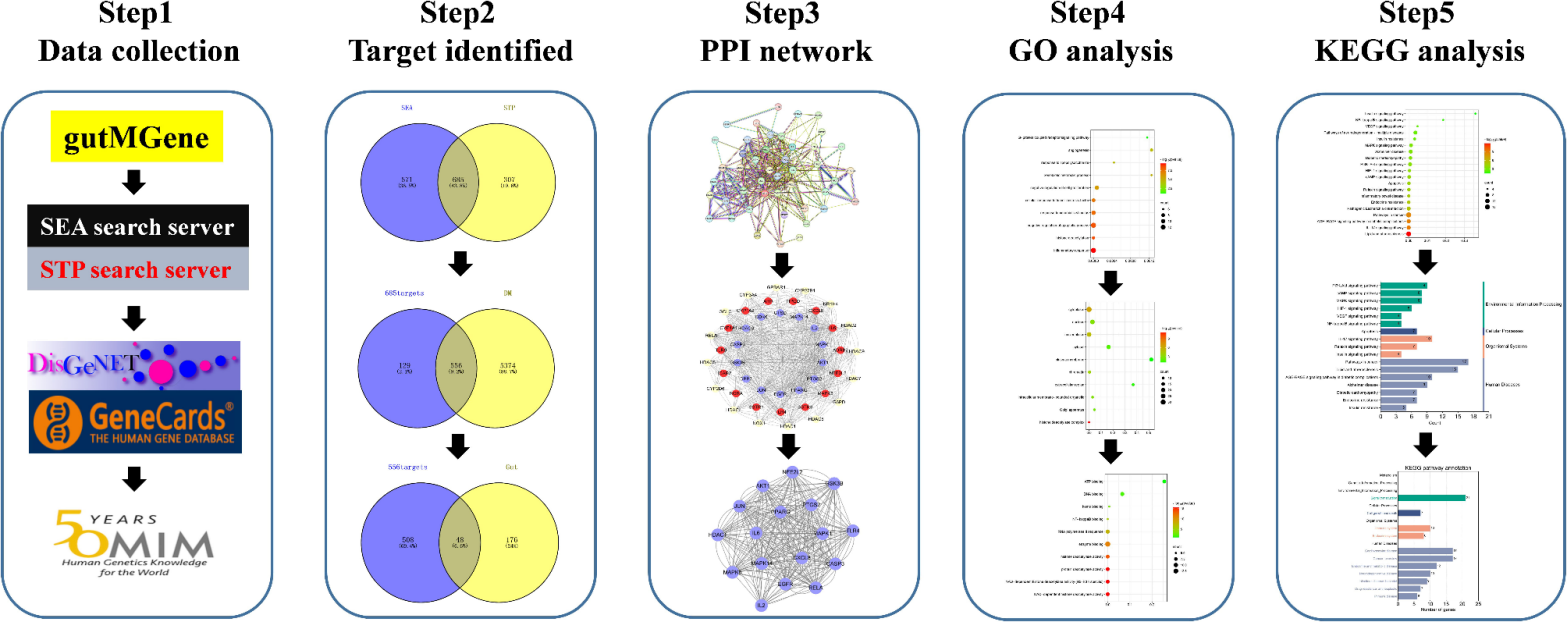
The flowchart reflecting our study design: Step 1: Data collection from open databases; Step 2: Identification of genes that regulate diabetes using VENN diagram; Step 3: Identification of hub genes using PPI network; Step 4: Identification of biological function of hub genes using GO function analysis; Step 5: Identification of major signaling pathway regulating DM by gut microbiota metabolites

We acquired the metabolites and human gut targets of gut microbiota from the gutMGene database. Subsequently, the metabolites were uploaded to the PubChem database to obtain their corresponding SMILES (simplified molecular input line entry system) format. The targets of the gut microbiota metabolites could be obtained from the SEA database and the STP database, with the species set as “homo sapiens”. By using the Venn diagram, the targets of gut microbiota metabolites from the SEA database and the STP database were intersected, which represents the common targets for further analysis. The database source and software used are displayed in Table 1, and Fig. 1 displays the flowchart of this study. Note: The method has some limitations. The gut microbiota data presented in the manuscript is derived from fecal samples (gutMGene database). Compared to biopsy samples, the microbial composition is varied from person to person, and it may not fully represent the real picture of microbial community in the gut. The data used in this manuscript is for network pharmacology prediction, more methods are need to further verify the accuracy of the gut microbiota data, including 16sRNA sequencing and fecal transplantation.

**Table 1:** Database, software and analysis platform

NO	Database, database and analysis platform	Website	Version
1	gutMGene	http://bio-annotation.cn/gutmgene/	\
2	Swiss Target Prediction	http: //www.swisstargetprediction.ch/	\
3	Pubchem	https://pubchem.ncbi.nlm.nih.gov/	\
4	bioinformatics	http://www.bioinformatics.com.cn/	\
5	STRING	https://string-db.org	V12.0
6	Cytoscape software	https://cytoscape.org/	V3.7.1
7	OMIM	https://omim.org/	\
8.	Genecards	https://www.genecards.org/	\
9	DisgeNet	https://www.disgenet.org/	\
10	DAVID Bioinformatics	https://david.ncifcrf.gov/tools.jsp	\
11	Similarity Ensemble Approach	https://sea.bkslab.org/	\
12	SwissAMDE	http://www.swissadme.ch/index.php	\
13	ADMETlab	https://admetmesh.scbdd.com/	\

### Identification of disease targets

Using “diabetes” as the keyword, we search for diabetes-related disease targets through the GeneCards database, DisGeNET and OMIM databases (The URL of disease database is available in Table 1). The targets from the GeneCards database that meet the criteria of Relevance score ≥ 10 were included in the analysis. Targets that appear twice were identified as diabetes common targets by using the Venn diagram.

### PPI network construction and analysis

The intersecting proteins of gut microbiota metabolites and DM key targets were uploaded to the STRING database to construct the Protein-Protein Interaction (PPI) network (The URL of STRING is available in Table 1).

### GO and KEGG enrichment analysis

The intersecting proteins were uploaded to the DAVID database to perform GO and KEGG enrichment analysis (The URL of DAVID is available in Table 1). The results were visualized using the bioinformatics platform. The statistics analysis: GO and KEGG terms that met the criteria “P < 0.05” were included for further analysis. Subsequently, “FDR (False Discovery Rate) < 0.05” was employed to identify the significant GO and KEGG terms.

### The evaluation of drug-likeness and toxicity

The identified key metabolites were measured for their drug-likeness and toxicity through the SwissAMDE platform and ADMETlab platform, respectively (The URL of SwissAMDE platform and ADMETlab platform are available in Table 1). By applying Lipinski’s Rule, researchers can conduct preliminary evaluations of molecules at the early stages of drug design and screen candidate drug molecules with high potential. This approach aids in saving time and resources, thereby enhancing the efficiency of drug development. The drug-likeness is determined based on the Lipinski’s rule of five: (1) molecular weight < 500; (2) lipid-water partition coefficient < 5; (3) hydrogen bond acceptor count < 5; (4) hydrogen bond donor count < 5; (5) polar surface area < 140. Metabolites that meet the criteria of Lipinski’s rule were selected for further analysis.

## Results

### The identifications of targets of gut microbiota metabolites intervening DM

**Fig. 2:**
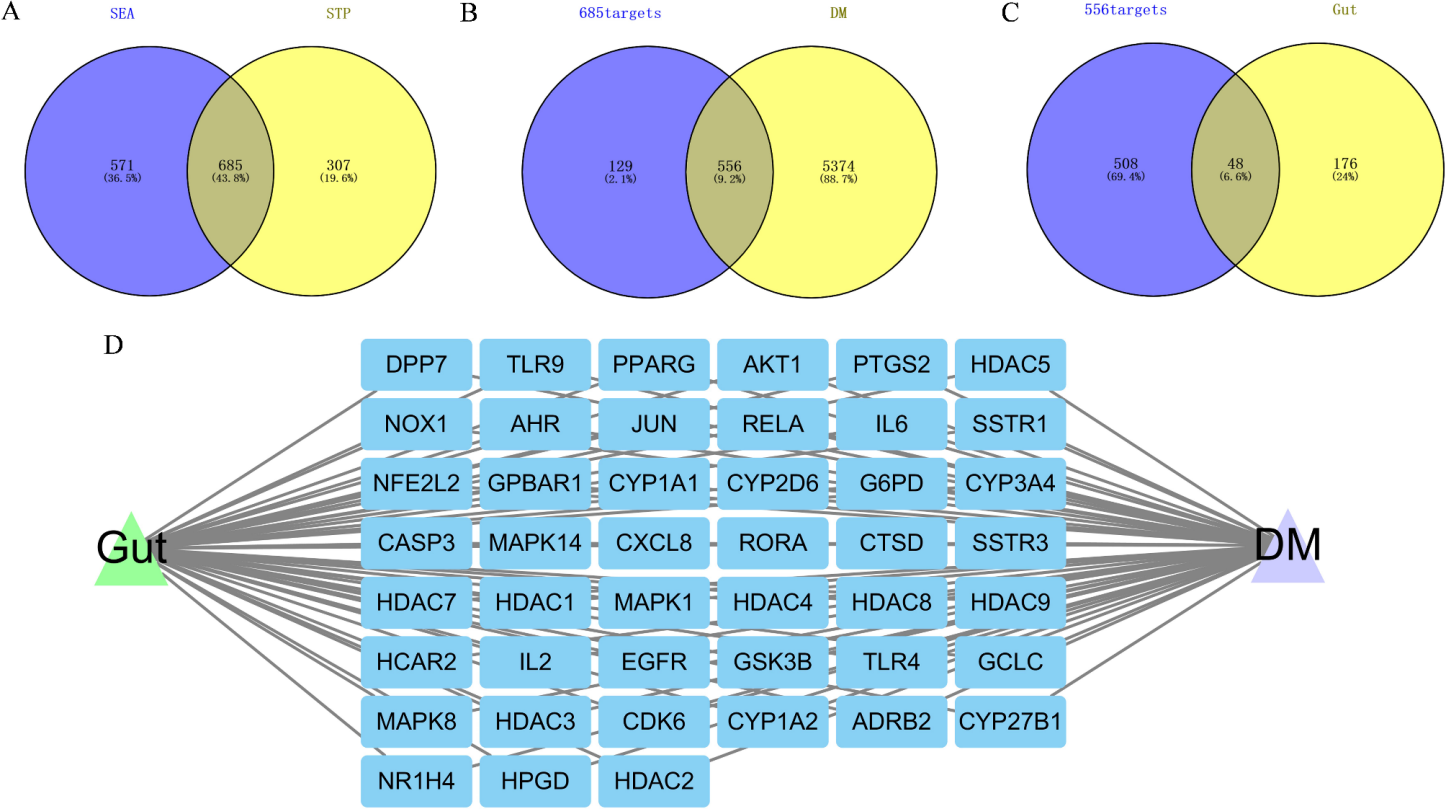
Identification of genes regulating DM by gut microbiota metabolites. (**A**) The common targets corresponding to gut microbiota metabolites between SEA and STP. (**B**) The common targets between targets of gut microbiota metabolites and DM. (**C**) The common targets between gut microbiota metabolites-DM and human gut targets. (**D**) The network of Gut-Target-DM. Note: SEA stands for similarity ensemble approach; STP stands for swiss target prediction; DM stands for diabetes mellitus

Firstly, we got 208 gut microbiota metabolites and 224 human gut targets from the gutMGene database. Then, we identified 1256 and 992 targets that are associated with 208 gut microbiota metabolites in the SEA and STP databases. The 685-overlapping targets between SEA and STP were considered the main targets of 208 gut microbiota metabolites (Fig. 2A). We retrieved a total of 5930 diabetes-related targets from the DisGeNET and OMIM databases. After intersecting the 685 gut microbiota metabolite common targets with these diabetes-related targets, we obtained 556 common targets (Fig. 2B). Lastly, we obtained a total of 48 targets from the VENN diagram between 556 common targets and gut targets (Fig. 2C). Figure 2D reflected the Gut-Targets-DM network. These results indicated that 48 targets are responsible for the regulation of DM by gut microbiota.

### PPI network construction

**Fig. 3:**
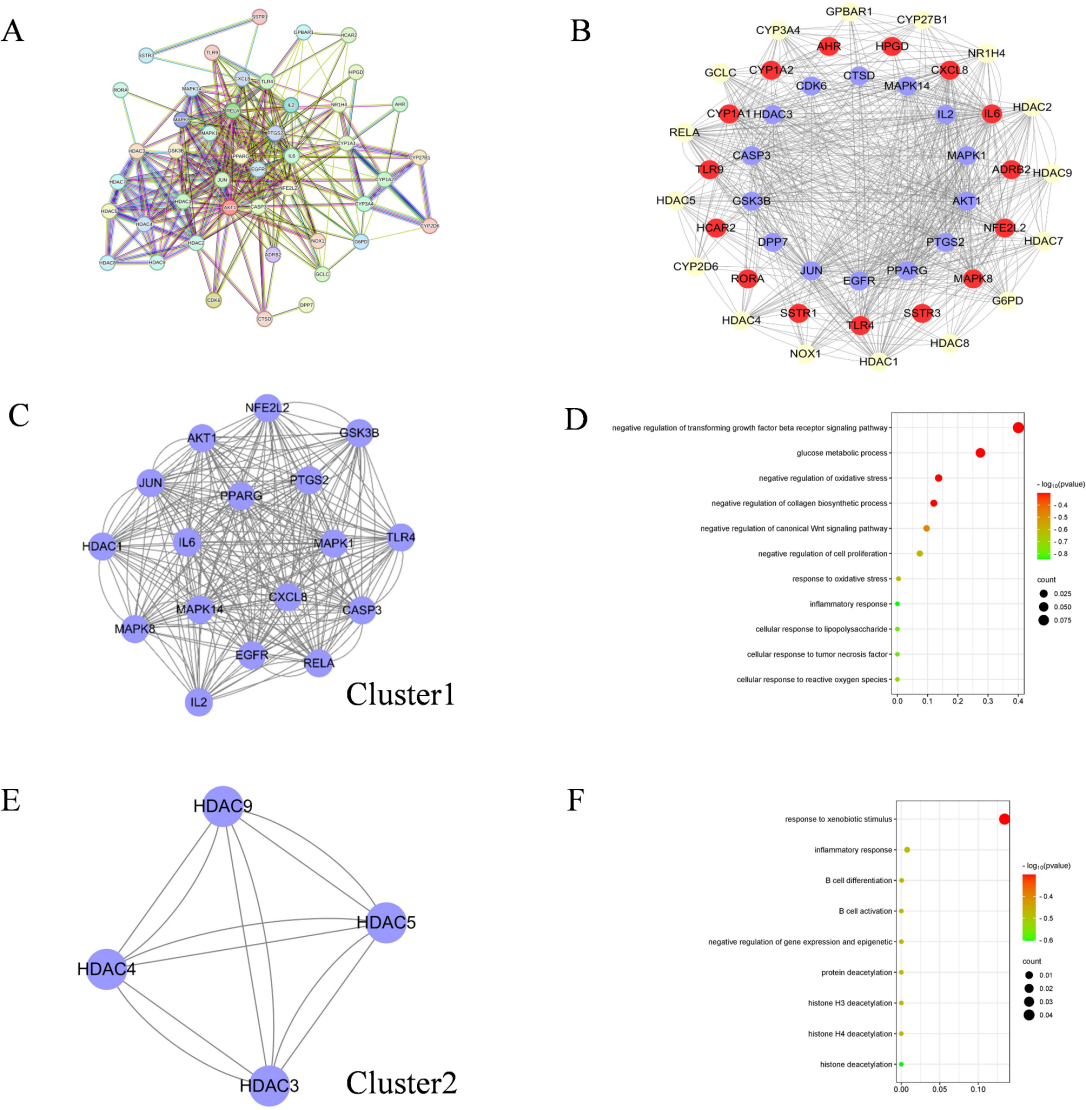
The enrichment analysis of PPI network. (**A**) The PPI network from STRING. (**B**) The visualization of PPI network. (**C**) The Cluster1 of PPI. (**D**) The GO-BP analysis of Cluster1. (**E**) The Cluster2 of PPI. (**F**) The GO-BP analysis of Cluster2

The overlapping targets were submitted to the STRING platform for PPI network analysis (Fig. 3A). And the network was visualized by Cytoscape 3.6, a total of 45 nodes and 612 edges were identified in the network (Fig. 3B). To further identify the core targets of gut microbiota metabolites in regulating diabetes, we conducted an in-depth analysis of the centrality of the targets using the Cytoscape plugin CytoNCA. The results showed that AKT1, IL-6, and PPARG had the highest DC (Degree Centrality) values (Table 2). We selected AKT1, IL-6, and PPARG as the core targets for gut microbiota metabolite regulation of diabetes based on their highest DC values.

To gain further insights into the functional significance of these targets, we employed the MCODE plugin to perform cluster analysis. As depicted in Fig. 3C and E, Cluster 1 has 17 nodes and 262 edges with a score of 16.374, Cluster 2 has 4 nodes and 12 edges with a score of 4. We also perform GO-BP analysis of Cluster 1 and Cluster 2. The x-axis of 3D and 3F represented the width of the chart. As depicted in Fig. 3D, the proteins in Cluster 1were mainly involved in the negative regulation of the TGF-β receptor signaling pathway and oxidative stress as well as inflammatory response. While proteins in Cluster 2 (Fig. 3F) were mainly responsible for histone deacetylation. The results implied that the function of PPI was mainly related to the negative regulation of the TGF-β receptor signaling pathway and oxidative stress as well as inflammatory response, which played a promoted role in the development of diabetes.

### GO enrichment analysis

**Fig. 4:**
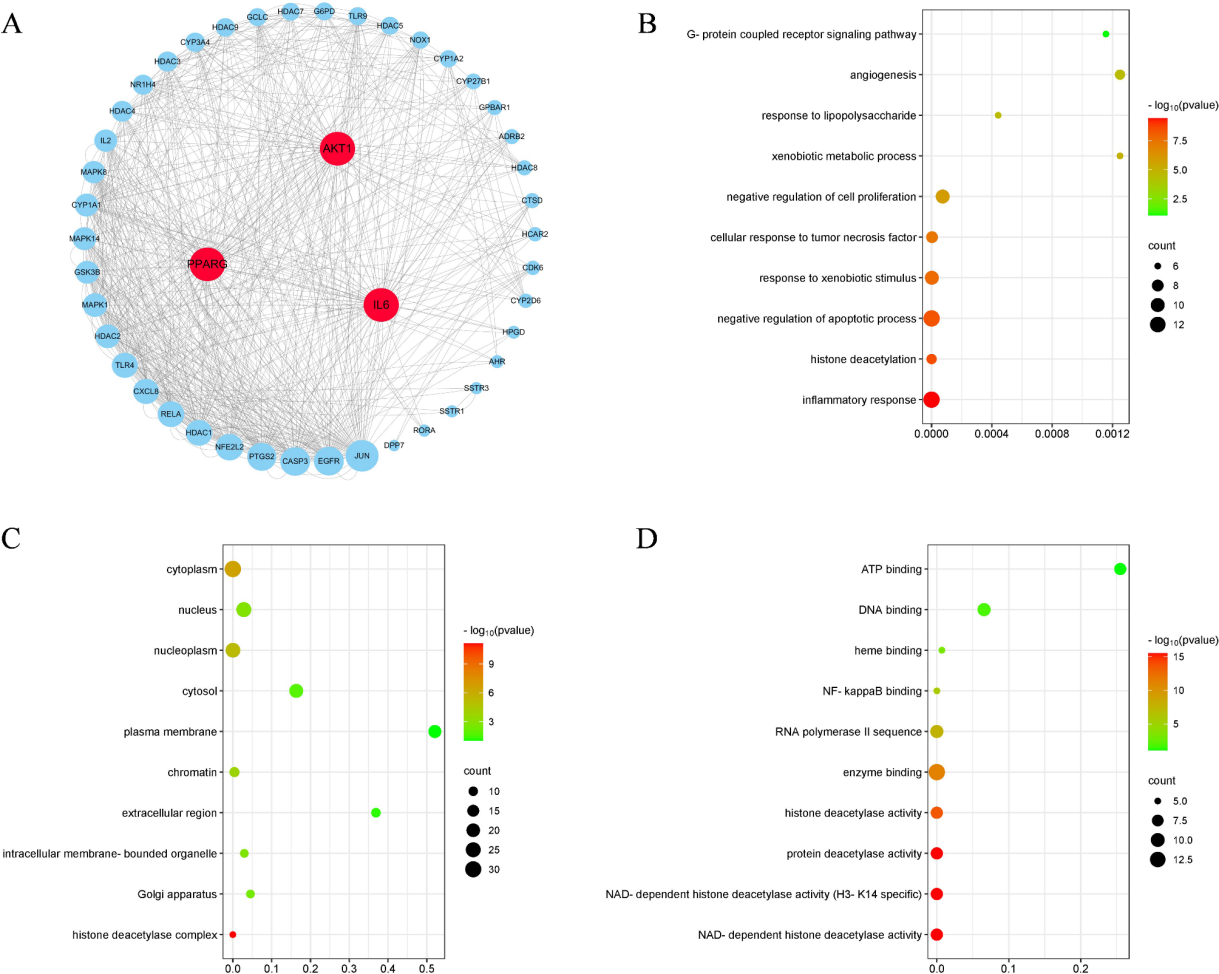
The GO function enrichment analysis of core targets of gut microbiota metabolites regulating DM. (**A**) The hub genes of PPI network. (**B**) The BP analysis of hub genes. (**C**) The CC analysis of hub genes. (**D**) The MF analysis of hub genes

GO analysis is a crucial tool that aids in describing the roles played by genes and proteins within cells. Figure 4A reflects the core hub gene that regulates DM. GO analysis results have revealed the top 5 BPs (Biological Processes) that are intricately linked to the treatment of diabetes mellitus (DM) through gut microbiota metabolites, as outlined below (Fig. 4B): inflammatory response, negative regulation of apoptotic process and cell proliferation, response to xenobiotic stimulus and tumor necrosis factor based on their gene counts. The top 5 CCs (Cellular Component) were (Fig. 4C) cytoplasm, nucleus, nucleoplasm, cytosol and plasma membrane. The top 5 MFs (Molecular Function) were (Fig. 4D) closely related to enzyme binding, RNA polymerase II sequence, DNA binding, NAD-dependent histone deacetylase activity and protein deacetylase activity. These results suggested the core target could influence the onset of diabetes by regulating inflammatory response, apoptotic process and activation of histone deacetylase.

### KEGG enrichment analysis

**Fig. 5:**
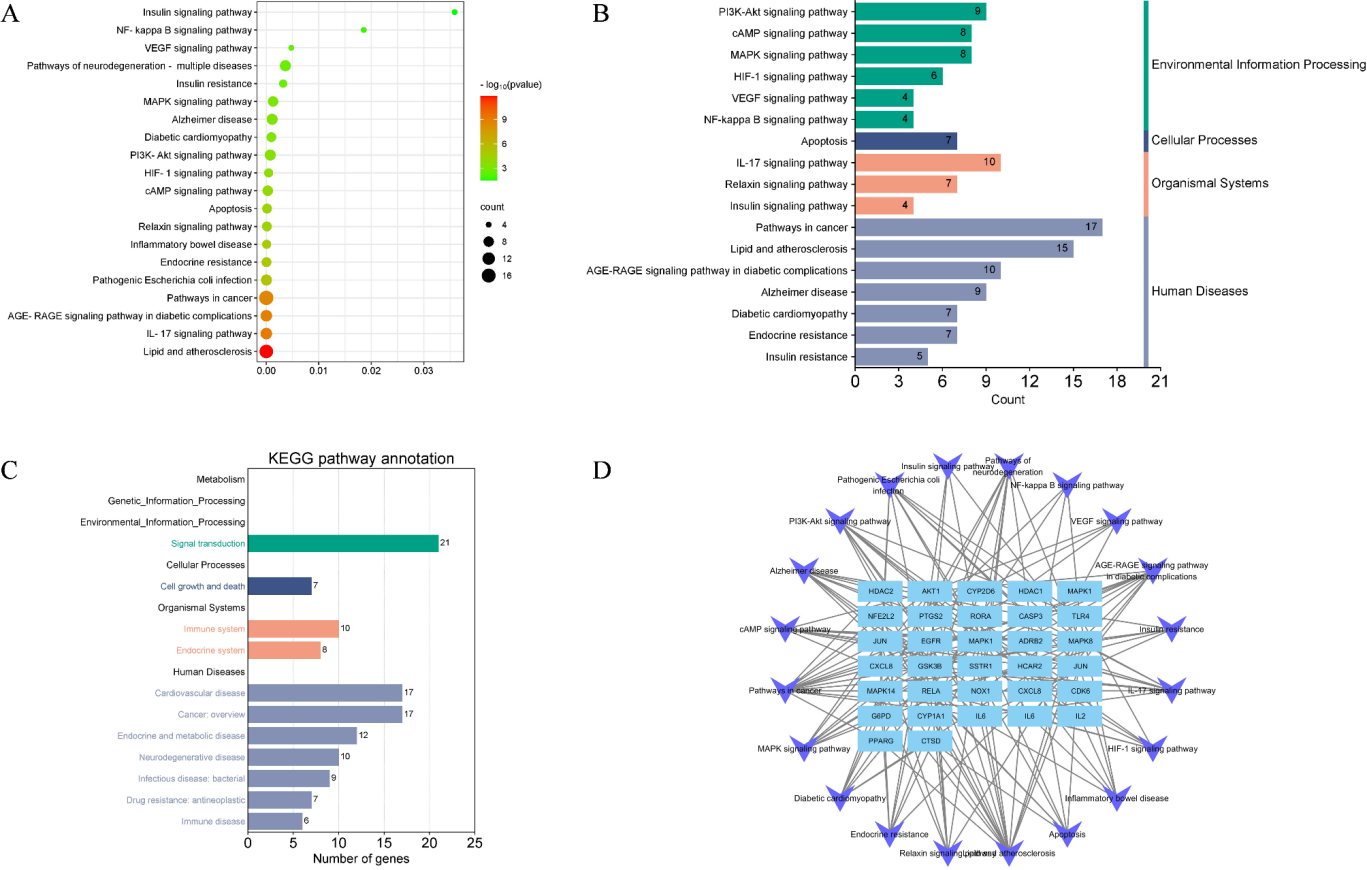
The KEGG enrichment analysis identified the core pathways of gut microbiota metabolites regulating DM. (**A**) KEGG pathway analysis. (**B**) KEGG classification analysis. (**C**) KEGG pathway annotation analysis. (**D**) The network of targets-pathways (Note: the V shape represents pathway, the blue rectangle represents target)

KEGG analysis is a powerful tool that enables the description of well-characterized biological pathways, as well as the prediction of the functions of genes or proteins that are currently unknown. This approach offers crucial insights into the regulatory mechanisms that govern biological processes, thus aiding us in their comprehensive understanding. The gut microbiota metabolites exerting protection against DM were mainly involved in the following major signaling pathways: AGE-RAGE pathway, IL-17 pathway, HIF-1 pathway, VEGF signaling pathway, NF-kappa B pathway, PI3K/Akt pathway, etc. (Fig. 5A). The KEGG enrichment analysis could be categorized into following aspects (Fig. 5B and C): Human Disease (Pathways in cancer, Lipid and atherosclerosis, Diabetic cardiomyopathy, Alzheimer disease and Endocrine resistance), Organismal systems (Immune system and Endocrine system), and Signal transduction (cAMP pathway, MAPK pathway, HIF-1 pathway, VEGF pathway, NF-kappa B pathway, PI3K/Akt pathway). Figure 5D displays the relationship network of pathways-targets, the purple color represents the signaling pathway and the blue color represents targets. Targeting the PI3K/Akt pathway and other inflammation- related pathway could attenuate the development of diabetes.

### The evaluation of drug-likeness and toxicity

We further evaluated the drug similarity and toxicity of the main metabolites in order to ensure their safety for therapeutic use. As shown in Table 2, Indole, 3-Indolepropionic acid, Trimethylamine oxide, Butyrate, Acetate, Propionate, Equol, 10-Keto-12Z-octadecenoic acid and other main metabolites all comply with the Lipinski’s Rule of Five (Table 2). Toxicity testing is a necessary process for drugs entering clinical treatment. Toxicity testing suggested (Table 4) that Indole, 3-Indolepropionic acid, Trimethylamine oxide, Butyrate, Acetate, Propionate, Equol and 10-Keto-12Z-octadec enoic acid do not cause liver damage or carcinogenicity. These results suggested that metabolites like Butyrate, Acetate and Propionate are beneficial to the gut health.

**Table 2:** The evaluation of drug-likeness properties on key metabolites

NO.	Target	DC value	NO.	Target	DC value
1	AKT1	64	24	HDAC9	22
2	IL6	64	25	HDAC7	20
3	PPARG	64	26	GCLC	20
4	JUN	58	27	G6PD	20
5	CASP3	50	28	TLR9	20
6	EGFR	50	29	HDAC5	20
7	PTGS2	48	30	NOX1	18
8	NFE2L2	44	31	CYP1A2	18
9	HDAC1	42	32	CYP27B1	14
10	RELA	42	33	GPBAR1	12
11	TLR4	40	34	HDAC8	12
12	CXCL8	40	35	CTSD	12
13	HDAC2	36	36	ADRB2	12
14	MAPK1	36	37	HCAR2	10
15	GSK3B	34	38	CYP2D6	10
16	MAPK14	34	39	CDK6	10
17	CYP1A1	34	40	HPGD	6
18	MAPK8	32	41	AHR	6
19	IL2	32	42	RORA	4
20	HDAC4	28	43	SSTR3	4
21	NR1H4	26	44	SSTR1	4
22	HDAC3	26	45	DPP7	2
23	CYP3A4	24			

**Table 3:** The evaluation of toxicity on key metabolites

Compound	MW	HBA	HBD	MLog *P*	Lipinski’sviolations	Bioavailability score	TPSA
Indole	117.15	0	1	1.57	0	0.55	15.79
3-Indolepropionic acid	189.21	2	2	1.4	0	0.85	53.09
Trimethylamine oxide	75.11	1	0	-1.66	0	0.55	29.43
Butyrate	87.1	2	0	0.49	0	0.85	40.13
Acetate	59.04	2	0	-0.49	0	0.85	40.13
Propionate	73.07	2	0	0.03	0	0.85	40.13
Equol	242.27	3	2	2.2	0	0.55	49.69
10-Keto-12Z-octadecenoic acid	296.44	3	1	3.59	0	0.85	54.37

Note: MW: molecular weight < 500; HBA: hydrogen bond acceptor < 10; HBD: hydrogen bond donor ≤ 5; MLog P: Moriguchi octanol-water partition coefficient ≤ 4.15; Lipinski’sviolations ≤ 1; Bioavailability score > 0.1; TPSA: topological polar surface area < 140

**Table 4:** The evaluation of toxicity on key metabolites

Compound	hERG Blockers	H-HT	DILI	Carcinogencity	LD50
Indole	Non-blocker	negative	negative	negative	5.328 mg/kg
3-Indolepropionic acid	Non-blocker	negative	negative	negative	4.475 mg/kg
Trimethylamine oxide	Non-blocker	positive	negative	negative	1.887 mg/kg
Butyrate	Non-blocker	positive	negative	negative	3.301 mg/kg
Acetate	Non-blocker	positive	negative	negative	2.597 mg/kg
Propionate	Non-blocker	positive	negative	negative	3.158 mg/kg
Equol	Non-blocker	negative	negative	negative	5.253 mg/kg
10-Keto-12Z-octadecenoic acid	Non-blocker	negative	negative	negative	3.792 mg/kg

Note: H-HT: Human Hepatotoxicity; DILI: Drug Induced Liver Injury; -

### The “M-S-M-T” network analysis

**Fig. 6:**
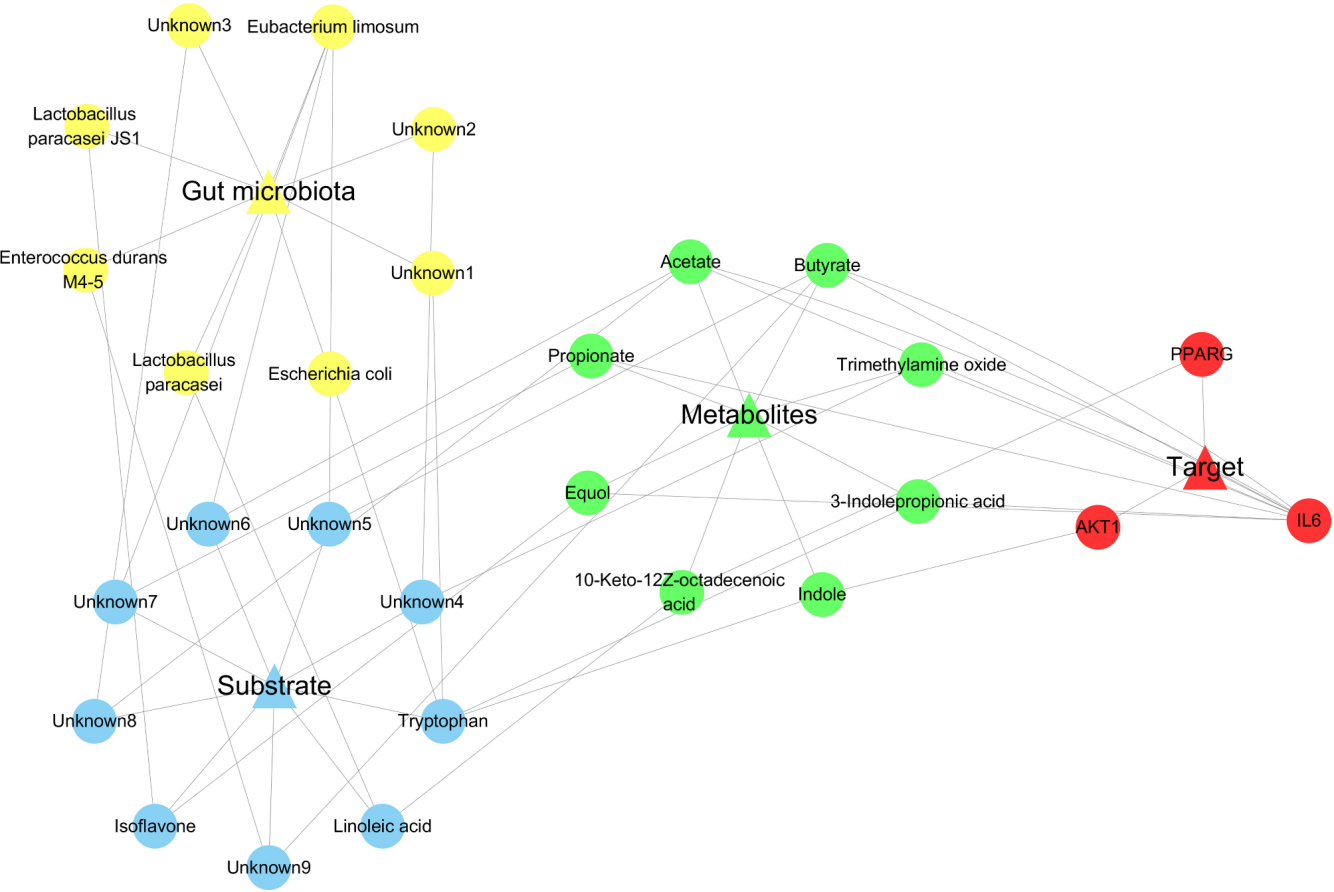
The network of Microbiota-Substrate-Metabolites-Targets. Note: The yellow color represents Gut microbiota, the blue color represents Substrate, the green color represents metabolites and the red color represents targets

We constructed a “Microbiota-Substrate-Metabolites-Targets” (M-S-M-T) network to elucidate their intricate relationship. We identified 8 metabolites, 9 substrates and 8 gut microbiotas associated with the 3 core targets (AKT1, IL-6, and PPARG) regulating DM. We employed Cytoscape 3.6 to visualise the network of M-S-M-T. As shown in Fig. 6, The yellow color represents Gut microbiota, the blue color represents Substrate, the green color represents Metabolites and the red color represents Targets. The AKT1 had 1 metabolite (Indole), 1 substrate (Tryptophan) and 1 gut microbiota (Escherichia coli). Although IL-6 had the most connection with metabolites and gut microbiotas, it possessed 7 unknown substrates and 3 unknown gut microbiotas. The PPARG had 1 metabolite (10-Keto-12Z-octadecenoic acid), 1 substrate (Linoleic acid) and 1 gut microbiota (Lactobacillus paracasei).

## Discussion

Diabetes has emerged as a significant global health concern, with its associated complications, notably diabetic nephropathy, serving as the primary etiology of end-stage renal disease (ESRD) (Navaneethan et al. 2023). Diabetes exerts a significant detrimental impact on patients’ quality of life, which not only generates huge treatment expenses but also engenders prolonged work incapacity among patients, thereby imposing a substantial burden on the healthcare system ((2023) 2023; Sarfo et al. 2023). Consequently, seeking efficacious therapies to mitigate the adverse consequences of diabetes is a top priority. We are going to use network pharmacology methodologies to comprehensively elucidate the intricate interplay between the “microbiota-substrate-metabolite-target” network in the regulation of diabetes.

Recently reported, a promising treatment is to adjust the quantity and composition of intestinal flora, which is expected to become a new strategy for the treatment of diabetes (Iatcu et al. 2021). Eating healthily and routine exercise are recommended as a major method to improve the gut microbial ecosystem (Khan et al. 2020). Compared with sedentary people, the abundance of beneficial microbiota such as *Roseburia*, *Akkermansia* and *Faecalibacterium* in regularly exercising people were increased (Mohr et al. 2020). Adapting a suitable diet style such as a Mediterranean diet and other food (such as green tea, yoga or nuts) can help restore the altered bacterial composition in the intestine. For obese patients, low-calorie diets can re-adjust the Firmicutes/Bacteroidetes ratio.

Furthermore, it has been observed that a diet rich in fiber is positively correlated with the elevated production of beneficial bacteria, while a diet high in protein is associated with an increase in the abundance of pathogenic bacteria (Jiang et al. 2022). In a study involving six obese patients with type 2 diabetes and/or hypertension, significant improvements were observed after adhering to a strict vegetarian diet for one month. These improvements included weight loss, and enhancements in fasting and postprandial blood glucose levels, the mechanism of these beneficial results is attributed to downregulating HbA1c and triglyceride levels (Dong et al. 2020). The dietary intervention was also found to be associated with a decrease in the Firmicutes/Bacteroidetes ratio and an increase in the abundance of Clostridium and Bacteroides fragilis, which in turn contributed to a reduction in gut inflammation and an elevation in short-chain fatty acid (SCFA) levels (Dowis and Banga 2021).

Pharmacology network analysis is a potent approach that utilizes advanced bioinformatics techniques to conduct systematic and comprehensive analyses of diverse biological molecules, thereby elucidating their intricate interactions and regulatory mechanisms (Nogales et al. 2022). By exploring various facets, including targets, pathways, drugs, and diseases, within pharmacological networks, researchers can acquire a holistic understanding of the intricate dynamics and complexity inherent in biological systems (Jiashuo et al. 2022). To investigate pivotal targets and metabolites for diabetes treatment, a combination of public databases and the construction of a “disease-gene-gut microbiota-metabolites” interaction network was employed, facilitating the identification of key targets and metabolites associated with diabetes.

The PPI network indicated AKT1, IL-6 and PPARG as hub genes that are involved in the mechanism of diabetes. AKT1 plays a pivotal role in the pathogenesis of diabetes, influencing not only glucose metabolism but also fatty acid homeostasis. Its activation can limit the catabolism of fatty acids, thereby maintaining lipid balance and preventing excessive lipid accumulation (Ramasubbu and Devi Rajeswari 2023). Additionally, AKT1 suppresses hepatic glucose output by inhibiting gluconeogenesis, which lowers blood glucose levels. AKT1 also promotes pancreatic beta cell survival, protecting them from apoptosis and preserving their functional integrity (Alwhaibi et al. 2019). However, diabetes contributes to dysregulated AKT1 activity that leads to pancreatic beta cell apoptosis and insulin resistance, promoting metabolic deregulation and disease progression.

IL-6, a multifunctional cytokine, exerts diverse biological effects. It has been implicated in the disruption of insulin receptor signaling, thereby promoting insulin resistance and hyperglycemia (Gal and Burchell 2023). Furthermore, IL-6 has been shown to directly impair pancreatic beta cells, thereby contributing to the pathogenesis and progression of diabetes (Cornwell et al. 2023). In adipocytes, IL-6 can activate inflammatory signaling pathways, leading to the secretion of pro-inflammatory mediators such as high-sensitivity C-reactive protein (hs-CRP) and IL-6 itself(Rose-John et al. 2023). These inflammatory factors have been implicated in the induction of insulin resistance, impairing the normal cellular glucose metabolism and precipitating the onset of diabetes(Wu et al. 2023a, b). Additionally, IL-6 has been found to modulate the secretory function of pancreatic beta cells, which are responsible for insulin production (Yu et al. 2023). By inhibiting the growth and secretory capacity of these cells, IL-6 could lead to insufficient insulin secretion. Consequently, this dysregulation may contribute to elevated blood glucose levels and exacerbate the clinical manifestations of diabetes.

PPARG, a member of the nuclear hormone receptor superfamily, plays a crucial role in regulating adipocyte differentiation and energy metabolism. Its activation has been shown to enhance the sensitivity of adipocytes and muscle cells to insulin, resulting in increased glucose uptake and utilization (Chen et al. 2023a, b). This mechanism contributes to the reduction of blood glucose levels and the alleviation of insulin resistance. Moreover, PPARG activation promotes adipocyte differentiation, leading to an augmented capacity for fat storage (Li et al. 2023). This effect is associated with a decrease in adipose tissue inflammation and oxidative stress, which further improves insulin resistance and glucose metabolism. Additionally, PPARG activation inhibits hepatic gluconeogenesis, thereby reducing the hepatic glucose output. Consequently, this action mode contributes to the reduced blood glucose levels and improvement in insulin resistance.

The KEGG enrichment results indicated that VEGF pathway, NF-кB pathway and PI3K/Akt pathway were closely involved in the development of diabetes. The PI3K/Akt pathway plays a pivotal role in regulating apoptosis, oxidative stress and inflammation, and it is closely associated with mesangial matrix proliferation and podocyte injury (Taheri et al. 2024). PI3K/Akt pathway has been confirmed as a crucial pathway in the pathogenesis of DM. Blocking the PI3K/Akt pathway could effectively ameliorate mesangial proliferation and reduce proteinuria (Aierken et al. 2022). Diabetic retinopathy is a serious complication of DM. High expression of VEGF was found in patients with DM, inhibiting the VEGF pathway could attenuate diabetes-induced retinal injury (Arrigo et al. 2022). Furthermore, the PI3K/Akt pathway serves as a vital insulin signaling molecule within the human body exerting significant regulatory effects on glucose and lipid metabolism (Xiao et al. 2022). Autophagy exerts renal protective effect by degrading unnecessary substances. PI3K/Akt pathway also modulates autophagy by regulating mTOR activity. Rapamycin could alleviate lipid accumulation and epithelial-to-mesenchymal transition (EMT) by enhancing autophagy activity via inhibiting the PI3K/AKT/mTOR signaling pathway (Jin et al. 2022). NF-кB pathway is a key inflammation regulator that participates in the development of DM. Under high glucose stimulation, the activated PI3K/AKT pathway can facilitate the dissociation of Iκb kinase from NF-κB, thereby releasing NF-κB and enabling its entry into the nucleus (Feng et al. 2023). This process promotes the transcription of relevant inflammatory cytokines, ultimately leading to the activation of the NF-κB pathway.

The physiological states are believed to have an important impact on the metabolic production. Based on the evaluation of drug-likeness and toxicity and potential application in the treatment of DM, we identified that butyrate, acetate and propionate are important metabolites derived from gut microbiota, which play a contributed role in regulating DM. Butyrate, acetate and propionate belong to SCFAs that have a positive effect on regulating glucose and lipid metabolism by improving insulin sensitivity, mitigating inflammatory responses and balancing energy homeostasis. DM could induce the occurrence of neurodegenerative diseases, such as Alzheimer disease. A study revealed that the deletion of acetate-producing gut microbiota could aggravate the development of neurodegenerative disease in diabetic animals, the acetate supplementations could restore cognitive impairment by regulating diversity of gut microbiota composition(Zheng et al. 2021). Inflammation promotes Insulin resistance, which is a key trigger of DM. Butyrate could improve insulin sensitivity by attenuating inflammation(Huang et al. 2023). Propionate could also protect neurons from high glucose stimulation as acetate did by reducing apoptosis via regulating the PI3K/AKT pathway(Wu et al. 2022).

In addition, the *Lactobacillus paracasei* and Linoleic acid were relevant gut microbiota and substance in the “M-S-M-T” network (Fig. 6). Exploring the connection between Lactobacillus paracasei and Linoleic acid is crucial for the development of novel therapy against DM. Linoleic acid is mainly derived from Lactobacillus paracasei. Linoleic acid is belonged to the members of long-chain fatty acids with essential functions in health and disease. The immune system plays a pivotal role in modulating the growth of tumors, and alterations in the gut microbiota community can, to a certain extent, impact the stability of the immune system. A study revealed that linoleic acid could inhibit the development of tumor by positively regulating CD8^+^ T cell and protecting mitochondrial integrity (Nava Lauson et al. 2023). Nonalcoholic fatty liver disease affects one-fourth of the global population, and delaying the development of liver fibrosis is regarded as a pivotal strategy for prolonging patients’ life expectancy. Linoleic acid could reduce liver fibrosis through inhibiting TGF-β signaling pathway and reducing inflammatory cytokines (Kasahara et al. 2023). The cognitive impairment is a severe complication of DM, Linoleic acid could improve cognitive performance by targeting CYP450-sEH pathway (Anita et al. 2023). The connection between Lactobacillus paracasei and Linoleic acid could be manifested as follows: Linoleic acid is mainly derived from *Lactobacillus paracasei*. In other words, the consumption of *Lactobacillus paracasei* from milk leads to the production Linoleic acid (Temimi et al. 2022). The intake of *Lactobacillus paracasei* is beneficial for body health. The supplementation of Lactobacillus paracasei could significantly reduce hyperglycemia, protect pancreatic β-cell and reduced oxidative stress in DM rats (Zeng et al. 2023). Moreover, Lactobacillus paracasei treatment could exert anti-diabetic effect on DM animals by regulating gut microbiota and reducing toxicity injury (Zeng et al. 2021). Taken together, *Lactobacillus paracasei* and Linoleic acid exhibit a multitude of beneficial impacts on bodily health, encompassing the reduction of hyperglycemia, oxidative stress and inflammation.

Although this study discovered the potential of gut microbiota in the treatment of DM, limitations still exist, and further measures should be taken to make it more perfect: (Wang et al. 2023) This study is based on data collection, and experimental validation should carry out in the future study to ensure its accuracy (Poulsen et al. 2023). The role of gut microbiota and their metabolites in the complications of diabetic complications, such as diabetic nephropathy, should be further explored (Korbut et al. 2023). Other therapies, such as food diet, natural products and herb medicine, that could regulate the composition of gut microbiota and production of metabolites should be investigated in future studies. Taken together, the next step should focus on a combination of network pharmacology and experimental validation to better explore the role of gut microbiota in the regulation of DM.

## Conclusion

This study employed a network pharmacology approach to investigate the underlying mechanisms of gut microbiota metabolites in the management of diabetes. The results offer us a comprehensive understanding of the therapeutic effects of previously identified target proteins, including AKT1, IL6, PPARG, JUN, CASP3, EGFR, and PTGS2, in the context of diabetes intervention. Moreover, the study identified high-degree value target proteins (AKT, IL-6, PPARG) and associated signaling pathways. Notably, the IL-17 signaling pathway, among others, remains relatively unexplored, highlighting its potential as a promising avenue for future research.

## Data Availability

No datasets were generated or analysed during the current study.

## Abbreviations

DM: Diabetes Mellitus
GO: Gene Ontology
KEGG: Kyoto Encyclopedia of Genes and Genomes
SCFAs: Short-chain fatty acids
PPI: Protein-Protein Interaction
VEGF: Vascular endothelial growth factor
GM: Gut microbiota
NF-κB: Nuclear Factor kappa-B
HIF-1: Hypoxia Inducible Factor-1
IL-6: Interleukin-6
PPARG: Peroxisome Proliferator-Activated Receptor Gamma
EGFR: Epidermal Growth Factor Receptor
PI3K: Phosphatidylinositol 3-kinase
EMT: Epithelial-to-Mesenchymal Transition
